# Artificial intelligence in nutritional health: sustainable consumers’ right to quality of life with special reference to youth

**DOI:** 10.3389/fpubh.2026.1817284

**Published:** 2026-07-20

**Authors:** G. Indirapriyadarsini, Sireesha Guttapalam, Ramyasri Mogarala, Kalpeshkumar L. Guptha

**Affiliations:** 1 Department of Law, Sri Padmavati Mahila Visvavidyalayam (Women's University), Tirupati, Andhra Pradesh, India; 2 Department of Home Science, Sri Padmavati Mahila Visvavidyalayam (Women's University), Tirupati, Andhra Pradesh, India; 3 Department of Research and Development Cell, Sri Padmavati Mahila Visvavidyalayam (Women's University), Tirupati, Andhra Pradesh, India; 4 Former Faculty, Gujarat National Law University, Surat, Gujarat, India

**Keywords:** artificial intelligence, consumer protection, food safety regulations, knowledge-attitude-practice (KAP) gap, progressive web application (PWA), sustainable development goals, sustainable health, youth health

## Abstract

**Background:**

Maintaining optimal youth nutritional health is an urgent socio-economic imperative that underpins long-term human productivity and rights-based development. However, modern youth cohorts face unique dietary threats caused by the widespread availability of ultra-processed foods, targeted digital marketing, and complex food labeling protocols. Although Artificial Intelligence (AI) presents innovative avenues for personalized dietary profiling, existing systems remain largely technocentric and detached from statutory frameworks or behavioral realities.

**Objective:**

This study bridges this interdisciplinary divide by evaluating a rights-based, technology-driven framework to improve youth nutritional health. It aims to: (1) empirically evaluate the “Knowledge-Attitude-Practice” (KAP) gap linking statutory consumer rights to real-world eating habits; (2) present the engineering design of a non-commercial Progressive Web Application (PWA) built to translate legal safeguards into daily behavioral changes; and (3) triangulate these findings using data from youth surveys and expert legal and nutritional panels.

**Methods:**

Using a cross-sectional approach based on non-parametric power constraints, a validated survey instrument was completed by a target sample of Indian youth (*n* = 354, aged 15–25 years). Concurrently, data matrices were compiled from regional legal experts (*n* = 12) and public nutrition professionals (*n* = 12) to cross-verify structural bottlenecks. Group variances, demographic dependencies, and rank associations were analyzed using robust non-parametric tests, including One-Way ANOVA, Kruskal-Wallis (H), Welch’s t-test, and Kendall’s Tau (*τ*) correlation coefficients.

**Results:**

Inferential analysis revealed unexpected demographic trends: undergraduate status predicted significantly higher FSSAI safety awareness than post-graduate status (*p* = 0.0037), while subjective health ratings exhibited a non-linear relationship with household income (*p* = 0.0004), peaking in the lower-middle financial tier. Crucially, rank correlation testing revealed that the relationship between statutory knowledge and actual dietary actions is functionally non-existent (*τ* = −0.001). This near-zero correlation provides clear empirical proof of a pronounced Knowledge-Action Gap, confirming that passive legal literacy fails to influence food selection in modern environments.

**Conclusion:**

By framing automated behavioral interventions within the constitutional protections of Article 21 of the Constitution of India and the Consumer Protection Act, 2019, this study shows how the open-access PWA (nutrition-zb.pages.dev) can bridge this behavioral gap. This shifts the focus of consumer health informatics from basic self-tracking to a rights-based, systemic public health intervention.

## 1 Introduction

Youth nutritional health represents a compounding public health priority globally, accelerated by rapid dietary transitions, digitized food marketing, and the pervasive availability of ultra-processed foods (1, 2). India houses one of the world’s largest adolescent and young adult demographics, yet this cohort is disproportionately burdened by a “double malady” of persistent micronutrient deficiencies alongside escalating rates of juvenile obesity (3, 4). Because nutritional trajectories forged during these formative years profoundly govern long-term metabolic health, cognitive output, and overall socioeconomic productivity, optimizing youth dietaries is direct scaffolding for Sustainable Development Goals (SDGs) 2 (Zero Hunger), 3 (Good Health and Well-Being), and 12 (Responsible Consumption and Production) (2, 5).

In the contemporary state, food choice is no longer an isolated act of individual agency. It operates within complex regulatory and corporate ecosystems. In India, nutritional welfare is protected through robust statutory architectures, notably the Food Safety and Standards Act (FSSA), 2006, the Consumer Protection Act (CPA), 2019, and the National Food Security Act, 2013 (6, 7). Furthermore, progressive judicial interpretations of Article 21 of the Constitution of India have structurally expanded the fundamental “Right to Life” to explicitly encompass the right to clean, unadulterated, and nutritionally adequate food (8). These legal measures vest citizens with actionable rights to safe consumption and mandate institutional accountability against deceptive marketing or substandard manufacturing.

Despite this legal apparatus, public health metrics indicate a deep fracture between statutory rights and actual consumer behavior. This disconnect highlights a classic phenomenon known in behavioral science as the Knowledge-Attitude-Practice (KAP) Gap or the Knowledge-Action Gap (9, 10). Traditional public health interventions operate on the rational-actor assumption: providing individuals with legal literacy and nutritional education will naturally result in optimal dietary execution (1). However, empirical literature demonstrates that cognitive awareness is routinely overridden by environmental stressors, financial constraints, peer dynamics, and aggressive corporate marketing (11).

This is where Artificial Intelligence (AI) introduces transformative potential. By leveraging machine learning models, natural language processing, and personalized data streams, AI can translate static legal protections and abstract nutritional guidelines into continuous, context-aware behavioral prompts (2, 12). Unfortunately, existing digital health tools remain aggressively technocentric. They focus narrowly on calorie-counting metrics while ignoring institutional policy landscapes, consumer rights, or local food environments (13).

To systematically address these issues, this paper introduces an integrated, interdisciplinary framework that unifies Food Law, Nutritional Science, and Human-Centered Computing (AI). Rather than treating the development of a digital app and an empirical survey as separate entities, this paper positions a Progressive Web Application (PWA) as an active intervention designed specifically to close the KAP gap identified by our empirical data.

### 1.1 Stated objectives

To maintain rigorous scientific boundaries and internal coherence, the paper’s scope is strictly constrained to the following objectives:

- Theoretical Formulation: Establish a behavioral framework mapping how consumer rights literacy and nutritional metrics intersect to influence youth quality of life (9, 10).
- Technical Architecture: Detail the design, data schemas, and engineering specifications of an expert-validated, open-access PWA (nutrition-zb.pages.dev) engineered to translate food rules and macro-tracking into automated behavioral nudges.
- Empirical Validation: Execute a cross-sectional survey of 354 youth participants to quantify real-world gaps between regulatory knowledge, socioeconomic standing, and dietary choices.
- Interdisciplinary Triangulation: Statistically evaluate variables using non-parametric models to determine how demographic variations govern health outcomes, validating the deployment criteria for targeted AI-driven interventions.

## 2 Theoretical framework and review of literature

### 2.1 The knowledge-attitude-practice (KAP) gap in digital health

Understanding health-seeking behavior requires moving past the assumption that knowledge equals action. In nutritional literacy, the KAP framework posits that while Knowledge forms the foundation and shifts Attitudes, the transition to Practice is highly non-linear (10, 14). The food landscape is saturated with ultra-processed choices, which creates a cognitive bottleneck. Youth may possess passive knowledge regarding food laws (e.g., recognizing the FSSAI logo), yet active micro-choices remain captured by convenience, taste, and aggressive digital ad campaigns (1, 15). By introducing an AI intermediary, this framework suggests that automated systems act as a continuous bridge. Machine learning algorithms automate the cognitive work required to assess food options. This lets users apply their abstract “right to safe food” directly during real-time retail selections (2, 16).

### 2.2 Right to health, consumer protection, and statutory realities

The Indian legal landscape provides clear structural support for consumer welfare:

- The Food Safety and Standards Act (FSSA), 2006: Establishes science-based standards for articles of food to regulate their manufacture, storage, distribution, sale, and import, ensuring the availability of safe and wholesome food for human consumption (7).
- The Consumer Protection Act (CPA), 2019: Strengthens consumer rights by introducing stringent liabilities for misleading advertisements, establishing the Central Consumer Protection Authority (CCPA), and codifying rights to be protected against the marketing of goods that are hazardous to life and health (6).
- Article 21 of the Constitution: Serves as the constitutional anchor. The Supreme Court of India has repeatedly affirmed that the right to health and decent nutrition is an integral element of a dignified life, transforming food safety from a policy goal into a constitutional right (8).

The core issue is that these legal frameworks are reactive, depending on post-consumption grievance redressal or macroscopic market enforcement. This paper proposes that a rights-based AI architecture can make these protections proactive. It puts regulatory compliance checking directly into the hands of the young consumer at the point of sale.

## 3 System architecture: the progressive web application (PWA)

To address the operational gaps identified in youth nutritional literacy and legal empowerment, a specialized, open-access Progressive Web Application was developed and deployed at: https://nutrition-zb.pages.dev/. The system was engineered by a cross-functional team comprising legal scholars, nutrition experts, and software developers to ensure that the platform’s computational logic reflects established scientific and statutory criteria.

### 3.1 Core architecture and technical specifications

The application is built as a lightweight, mobile-responsive Progressive Web Application (PWA) leveraging modern web technologies (HTML5, advanced JavaScript frameworks, and localized CSS rendering engines). The platform runs client-side to ensure privacy, rapid rendering, and offline utility in low-connectivity environments, using automated Service Workers for asset caching and local indexing. The system architecture is structured across three distinct modules:

1. Nutritional Diagnostics Engine: Computes customized physical indices, including Basal Metabolic Rate (BMR) via the Mifflin-St Jeor equation and Total Daily Energy Expenditure (TDEE) based on user-inputted activity levels. It maps daily macronutrient (carbohydrates, proteins, fats) and micronutrient allowances against the standardized dietary guidelines formulated by the Indian Council of Medical Research-National Institute of Nutrition (ICMR-NIN) (4, 17).
2. Regulatory & Label Literacy Module: A structured educational interface that decodes ingredient lists and front-of-package labels. It alerts users to hidden ultra-processed chemical agents, excessive sodium, and trans fats, linking these directly to consumer protection guidelines under the FSSA (7).
3. Consumer Grievance System Matrix: Provides users with a clear, step-by-step pipeline for reporting food safety violations. It guides them from local Food Safety Officers up to the National Consumer Helpline and integrated FSSAI portals, making abstract consumer rights practically actionable (6).

### 3.2 Computational design features

The PWA deploys 28 interactive operational features designed to transform users from passive consumers into active, legally literate decision-makers:

- Dynamic Nutrient Tracking Sliders: Allows real-time visualization of accumulated macro-intake against daily recommended allowances.
- Static Legal Response Frameworks: Interactive decision-trees that allow users to select a food-related injury (e.g., contamination, adulteration, deceptive volume labeling) and instantly generate the specific legal grounds and appropriate jurisdiction for filing a complaint under the Consumer Protection Act, 2019 (6).
- Validated Data Infrastructure: The application contains no speculative or unverified third-party food databases. All food composition profiles, caloric densities, and mineral values are hard-coded from the verified Indian Food Composition Tables (IFCT) generated by ICMR-NIN (17). This ensures complete scientific reproducibility and protection against commercial algorithmic bias.

## 4 Empirical methodology

### 4.1 Participant recruitment and sampling strategy

To empirically evaluate the target population and validate the real-world requirement for this digital intervention, a cross-sectional study was executed. The target population was defined as Indian youth aged 15 to 25 years—a demographic chosen due to their unique behavioral, developmental, and economic vulnerability within modern digital food environments (15). The minimum sample size was determined using a non-parametric power analysis equation for large populations: n = (Z^2 * p * (1-p)) / e^2, where Z = 1.96 (95% Confidence Level), *p* = 0.5 (Expected prevalence maximizing required sample size to ensure statistical robustness across highly diverse socioeconomic variables), and e = + − 0.05 (Margin of error). The mathematical minimum sample requirement of 384 was modified to an operational target of 354 completed responses, providing a highly powered sample size to run robust inferential non-parametric comparisons.

Participants were recruited using a multi-channel sampling approach over a designated timeframe. To ensure geographic, socioeconomic, and cultural diversity, the survey instrument was distributed across higher education institutes, vocational training centers, and student residential complexes (hostels) within urban and semi-urban hubs. This dual-channel distribution approach balanced localized academic sampling with broad online accessibility to capture a representative cross-section of both institutionalized and non-institutionalized youth.

### 4.2 Inclusion and exclusion criteria

- Inclusion Criteria: Participants must be aged between 15–25 years at the time of data collection; must reside in India; must possess basic digital literacy to interact with smartphone-based surveys; and must provide explicit informed consent. For minors (under 18 years of age), parental/institutional consent was mandatory.
- Exclusion Criteria: Individuals with pre-existing, severe clinical eating disorders (e.g., Anorexia Nervosa, Bulimia) or severe cognitive or psychological impairments were excluded. This prevented confounding the baseline evaluation of routine consumer choice and legal literacy patterns.

### 4.3 Survey instrument design and validation

Data collection relied on a multi-dimensional, structured questionnaire systematically partitioned into five distinct thematic sections: Section 1 (Demographics) captures age, gender, educational level, monthly household income, and residential status. Section 2 (Nutritional Health & Dietary Habits) quantifies individual consumption behaviors, focusing on the frequency of junk food and ultra-processed packaging. Section 3 (Consumer Rights Literacy) assesses actionable awareness regarding the CPA 2019 (6). Section 4 (Food Safety Regulations & Public Health Trends) evaluates knowledge of FSSAI labeling mandates and includes an exploratory item tracking general concern regarding rising substance and drug abuse trends within their local youth demographic (7, 18). Section 5 (Subjective Quality of Life) measures self-rated physical well-being using standardized Likert scales.

To ensure structural reliability, the ad-hoc elements of the survey instrument were subjected to a formal pre-test and pilot study (*n* = 35). The internal consistency of the scaled items across Sections 3, 4, and 5 was statistically evaluated, yielding a global Cronbach’s Alpha coefficient (*α*) of 0.79. This confirms that the questionnaire meets established psychometric requirements for internal consistency before full-scale deployment.

### 4.4 Data cleaning, preparation, and imputation protocols

Raw survey records were exported into a structured electronic matrix. To guarantee high-fidelity data integrity, strict preprocessing steps were applied: (a) Any survey containing missing data in core demographic identifiers or more than 10% unrecorded responses across the scaled matrices was discarded; (b) For minor, isolated missing values in non-critical ordinal items (<2% of total dataset), a median imputation technique was applied; (c) Categorical metrics were assigned systematic code parameters to support robust group comparisons.

### 4.5 Statistical analysis strategy

The analytical framework was designed to evaluate non-normal distributions, ordinal rankings, and multi-group variances: Descriptive analysis (frequencies, percentages, central tendencies) was performed first. Group variances across multi-category demographic variables were evaluated using One-way Analysis of Variance (ANOVA) accompanied by robust Kruskal-Wallis (H) tests to protect the integrity of ordinal conclusions (19). Post-hoc pairwise comparisons were executed using Tukey’s HSD tests. To map the strength and directional vectors of inter-sectional dynamics, bivariate correlation matrices were computed using Kendall’s Tau (*τ*) coefficient over Pearson’s to protect against outlier distortion. Qualitative framework triangulation from expert panels (*n* = 24) was conducted via Thematic Analysis.

## 5 Results and discussion

### 5.1 Demographic composition of the sample cohort

The empirical profile of the target youth population (*n* = 354) outlines a crucial developmental stage characterized by transitioning autonomy over health behaviors. 57% of respondents fell within the 15–20 age bracket (primarily undergraduate tracks), while 43% were situated in the 21–25 age bracket (enrolled in post-graduate programs). The gender breakdown exhibits a higher representation of female respondents (78%) compared to male respondents (22%). Geographically, 65% of the participants were located in urban or semi-urban regions, with 35% tracking from rural backgrounds. Socioeconomically, the sample exhibits a pronounced low-to-middle income concentration: 57% of youth households earn less than ₹20,000 per month.

### 5.2 Bivariate group variations and demographic predictors

To test whether regulatory knowledge and subjective well-being are uniform across youth demographics, non-parametric inferential analyses were executed (19) (see Tables 1–3).

**Table 1:** Inferential group variance & demographic predictors (ANOVA and Kruskal-Wallis matrix).

Demographic variable	Survey section score monitored	Statistical test executed	Test value/*p*-value	Specific post-hoc pairwise finding
Education	S3: FSSAI Regulatory Awareness (Scale: 1–3)	One-Way ANOVA	p = 0.0037**	Undergraduate students show a significantly higher mean awareness score (2.48) than Post-Graduate students (2.21).
Income	S5: Overall Subjective Health (Scale: 1–4)	One-Way ANOVA	p = 0.0004***	The mid-low income bracket (₹10 k–₹20 k) records a significantly higher health score than extreme bands.
Gender	S4: Regional Drug Abuse Concern (Scale: 1–4)	Kruskal-Wallis	p = 0.0062**	Female respondents express a significantly higher median concern (Mdn = 4.0) than Male respondents (Mdn = 3.0).
Income	S5: Overall Subjective Health (Scale: 1–4)	Kruskal-Wallis	*p* = 0.0007***	Complete distribution patterns for self-rated health vary non-linearly across structural wage categories.

***p < 0.001, **p < 0.01.

**Table 2:** Complete cross-sectional demographics bivariate significance grid (*p*-values).

Demographic variable	S2: Junk food frequency	S3: Consumer rights awareness	S4: Food laws & drug concerns	S5: Perceived overall health
Age group	0.611	0.059	0.380	0.341
Education grouped	0.402	0.007**	0.042*	0.165
Gender	0.322	0.253	0.006**	0.774
Income	0.578	0.362	0.555	0.001***

**Table 3:** Bivariate correlation matrix (Kendall’s Tau-$\tau$ structural heatmap).

Dimension	S1: Demog	S2: Diet	S3: Rights	S4: Laws	S5: Health
S1: Demographics	1.000	−0.036	0.154	0.100	−0.074
S2: Nutritional habits	−0.036	1.000	−0.001	−0.008	−0.146
S3: Consumer rights	0.154	−0.001	1.000	0.162	0.146
S4: Food laws (NDPS)	0.100	−0.008	0.162	1.000	0.039
S5: Perceived health	−0.074	−0.146	0.146	0.039	1.000

The inferential variance mapping reveals two anomalies: First, higher educational tiers do not predict linear improvements in regulatory literacy. Undergraduates significantly outscored postgraduates on FSSAI parameters (*p* = 0.0037), highlighting that recent institutional integration or digital consumer interactions provide superior functional knowledge. Second, subjective health displays a non-linear relationship with income (*p* = 0.0004); lower-middle financial tiers record optimal health index scores, which structurally drop as income increases, matching a transition toward processed delivery and urban stressors (15).

### 5.3 Correlation analysis: quantifying the knowledge-action gap

To evaluate how behavioral dimensions interact, a multi-section correlation matrix was computed using Kendall’s Tau (*τ*) coefficients.

The load-bearing empirical result is clear: the rank correlation between active dietary execution (S2) and statutory consumer knowledge (S3/S4) is functionally non-existent (τ = −0.001 and τ = −0.008). This near-zero association represents mathematical verification of the KAP Gap (10). Knowing about FSSAI mandates or CPA mechanisms exercises no control over real-time youth purchasing behaviors, proving that passive legal education fails against commercial processing algorithms (1). In contrast, a clear, powered negative correlation connects fast-food frequency with subjective well-being (*τ* = −0.146), validating the requirement for automated point-of-sale interventions.

### 5.4 Contextual calibration: justification of macro-environmental variables

To preserve scientific focus, monitoring youth concern regarding broader lifestyle threats (e.g., substance/drug misuse under Section 4) functions as an analytical control checking if youth harbor a generalized health-conscious mindset (18). The data shows significant gender variation in risk perception (*p* = 0.0062), with females expressing elevated concern. Crucially, macro-lifestyle concerns demonstrate zero correlation with actual physical health outcomes (τ = 0.039), meaning that abstract environmental anxieties fail to govern concrete personal execution without automated tools.

### 5.5 Multi-stakeholder triangulation: convergence and divergence gaps

Triangulation between youth profiles and expert matrices (*n* = 24) exposed deep structural fractures: While 62% of youth claim acceptable health statuses, nutrition experts flatly reject this, noting that youth conflate shallow digital literacy with functional dietary management. Furthermore, legal experts emphasize that existing frameworks under the CPA 2019 are strictly reactive, proving the absolute necessity for real-time preventive tools like the PWA at the point of purchase.

### 5.6 Algorithmic justification: how the PWA directly closes the empirical gap

- Mitigating the KAP Gap: Since legal literacy exhibits a near-zero rank correlation with diet choices, the PWA shifts execution from memory-compliance to client-side automated processing (Mifflin-St Jeor and ICMR-NIN reference mappings).
- Proactive Legal Shielding: The platform’s Regulatory Module decodes hidden additives against FSSAI guidelines, instantly turning static rules into live point-of-sale consumer armor.
- Socioeconomic Accessibility: Targeting the 57% lower-income threshold group, the PWA functions entirely client-side on open-access, zero-cost parameters using verified IFCT tables (17).

## 6 Policy recommendations and institutional frameworks

To systematically close the verified Knowledge-Action Gap, public policy must undergo an evolution from passive educational disclosure toward automated, rights-based structural frameworks (9, 10).

### 6.1 Digital governance: mandating automated public health informatics

MeitY, in systemic alignment with the Ministry of Health and Family Welfare, should mandate the integration of non-commercial, open-source nutritional informatics frameworks within institutional public portals (2). Using the client-side architecture of the nutrition-zb.pages.dev model ensures comprehensive, privacy-preserving macronutrient mapping without third-party data tracking.

### 6.2 Structural enforcement: regulating educational and digital food environments

The FSSAI must aggressively enforce the Food Safety and Standards (Safe Food and Balanced Diets for Children in School) Regulations, creating total bans on high-fat, high-sodium (HFSS) items inside university and school zones (7). Concurrently, the CCPA must impose strict algorithmic guidelines under the CPA 2019 to suppress deceptive digital food-delivery advertisements targeting young demographics (6).

### 6.3 Statutory evolution: implementing dynamic front-of-package labeling (FOPL)

To insulate the 38% of hostel-dwelling youth vulnerable to dietary convenience, India should accelerate the implementation of interpretative, color-coded Front-of-Package Labeling (FOPL) systems (1). This transforms passive chemical descriptions into rapid, machine-readable indicators, enabling applications to parse risks instantly before transaction clearance.

### 6.4 Legal accountability: activating proactive grievance pipelines

We recommend embedding automated class-action generation modules directly within consumer interfaces. If a scanned item exposes an illegal stabilizer concentration violating Section 22 of the FSSA 2006, the platform should auto-compile and file a formal compliance report to the National Consumer Helpline, transforming passive statutory definitions into live mechanisms of developer accountability (6, 7).

## 7 Conclusion

This interdisciplinary research establishes a comprehensive framework bridging Food Law, Nutritional Science, and Artificial Intelligence. The empirical reality of 354 youth respondents reveals that standard legal consumer literacy holds a near-zero correlation with actual dietary choice (*τ* ~ 0.00), exposing a deep Knowledge-Action Gap driven by income parameters and ultra-processed market saturation. To systematically resolve this, the deployed open-access PWA (nutrition-zb.pages.dev) serves as an active technological intervention, executing client-side algorithmic calculations and regulatory compliance checks to insulate consumer choices at the point of sale. Ultimately, optimizing youth nutrition must transition from an individual lifestyle choice into an actively protected constitutional right under Article 21 of the Constitution of India, secured via automated, human-centered computing architectures.

## Data Availability

The original contributions presented in the study are included in the article/supplementary material, further inquiries can be directed to the corresponding author.
